# Cerebral Small Vessel Disease Burden Is Associated With Poststroke Depressive Symptoms: A 15-Month Prospective Study

**DOI:** 10.3389/fnagi.2018.00046

**Published:** 2018-02-28

**Authors:** Yan Liang, Yang-Kun Chen, Vincent Chung-Tong Mok, De-Feng Wang, Gabor S. Ungvari, Winnie Chiu-Wing Chu, Hee-Ju Kang, Wai-Kwong Tang

**Affiliations:** ^1^Department of Psychiatry, The Chinese University of Hong Kong, Hong Kong, Hong Kong; ^2^Department of Neurology, Dongguan People’s Hospital, Dongguan, China; ^3^Department of Medicine and Therapeutics, The Chinese University of Hong Kong, Hong Kong, Hong Kong; ^4^Department of Imaging and Interventional Radiology, The Chinese University of Hong Kong, Hong Kong, Hong Kong; ^5^Graylands Hospital, The University of Notre Dame Australia, Perth, WA, Australia; ^6^Department of Psychiatry, Chonnam National University Hospital, Gwangju, South Korea; ^7^Shenzhen Research Institute, The Chinese University of Hong Kong, Shenzhen, China

**Keywords:** depressive symptoms, stroke, poststroke depression, cerebral small vessel disease, white matter hyperintensities, lacune, cerebral microbleeds, enlarged perivascular spaces

## Abstract

**Objective:** All types of cerebral small vessel disease (SVD) markers including lacune, white matter hyperintensities (WMH), cerebral microbleeds, and perivascular spaces were found to be associated with poststroke depressive symptoms (PDS). This study explored whether the combination of the four markers constituting an overall SVD burden was associated with PDS.

**Methods:** A cohort of 563 patients with acute ischemic stroke were followed over a 15-month period after the index stroke. A score of ≥7 on the 15-item Geriatric Depression Scale was defined as clinically significant PDS. Scores of the four SVD markers ascertained on magnetic resonance imaging were summed up to represent total SVD burden. The association between SVD burden and PDS was assessed with generalized estimating equation models.

**Results:** The study sample had a mean age of 67.0 ± 10.2 years and mild-moderate stroke [National Institutes of Health Stroke Scale score: 3, interquartile, 1–5]. PDS were found in 18.3%, 11.6%, and 12.3% of the sample at 3, 9, and 15 months after stroke, respectively. After adjusting for demographic characteristics, vascular risk factors, social support, stroke severity, physical and cognitive functions, and size and locations of stroke, the SVD burden was associated with an increased risk of PDS [odds ratio = 1.30; 95% confidence interval = 1.07–1.58; *p* = 0.010]. Other significant predictors of PDS were time of assessment, female sex, smoking, number of acute infarcts, functional independence, and social support.

**Conclusion:** SVD burden was associated with PDS examined over a 15-month follow-up in patients with mild to moderate acute ischemic stroke.

## Introduction

Poststroke depression (PSD) is associated with higher risk of mortality, disability, and poor quality of life (Towfighi et al., 2017). About one in three stroke survivors present with PSD at any time after stroke (Towfighi et al., 2017). Understanding the underlying pathophysiology of PSD may facilitate developing better management strategies. Findings concerning the relationship between stroke locations and PSD have been inconsistent (Carson et al., 2000; Wei et al., 2015), which challenges the hypothesis that PSD is caused by a single lesion in the brain due to stroke (Stern and Bachman, 1991).

Cerebral small vessel disease (SVD) is a composite term for a variety of chronic cerebral microvascular lesions resulting from aging, vascular risk factors or unknown etiologies (Wardlaw et al., 2013). SVD is common in aging and stroke populations (Wardlaw et al., 2013). The most characteristic markers of SVD on magnetic resonance imaging (MRI) are WMH, lacune, cerebral microbleeds, and enlarged perivascular spaces (Wardlaw et al., 2013). A “SVD score” that includes the above four MRI markers was recently proposed to quantify the global damage to the small vessels of the brain caused by SVD (Staals et al., 2014). The SVD burden is associated with cognitive decline in hypertension (Uiterwijk et al., 2016), poststroke disability (Arba et al., 2017a), recurrence of stroke (Lau et al., 2017), poor quality of life of stroke survivors (Liang et al., 2017), and depression after lacunar stroke (Zhang X. et al., 2017).

Individual SVD markers have been shown to be associated with PSD (Santos et al., 2009a; Tang et al., 2014). Only one study has reported an association between SVD burden and poststroke depressive symptoms (PDS) 3 months after lacunar stroke (Zhang X. et al., 2017). However, the impact of SVD burden on the long-term prevalence of PSD has never been investigated. Similarly, the role of SVD burden in the development of PSD in a cohort comprising all subtypes of acute ischemic stroke is unclear. Hence, this study examined the association between SVD burden and poststroke depressive symptoms (PDS) during the first 15 months following an acute ischemic stroke.

## Materials and Methods

### Study Population

**FIGURE 1:**
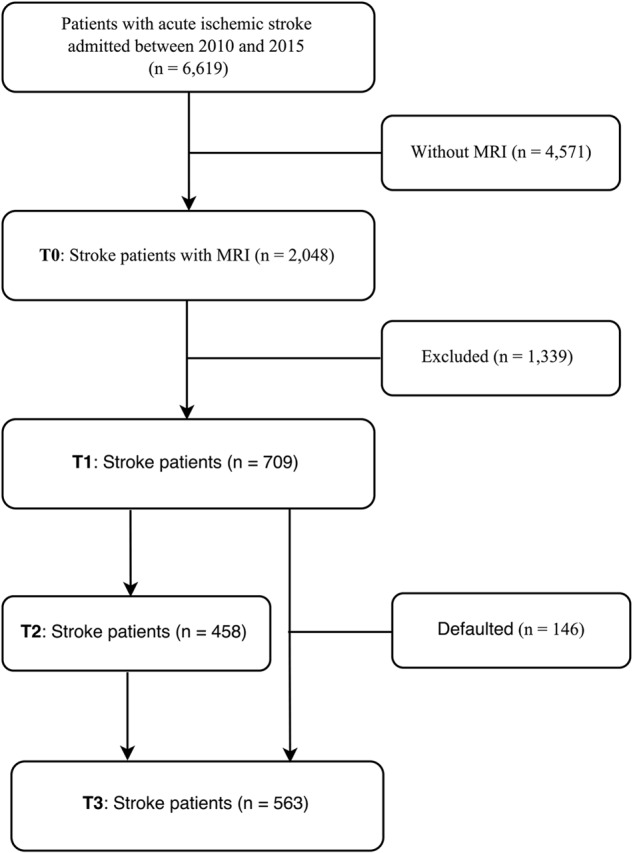
Flow chart of the study.

The recruitment of the study sample is depicted in **Figure 1** where T0–T3 refer to assessment points at baseline, 3, 9, and 15 months after the index stroke, respectively. Patients with first-ever or recurrent acute ischemic stroke were consecutively screened following their admission to the Stroke Unit, Prince of Wales Hospital, Hong Kong, between January 2010 and October 2015. Due to the limited access to MRI, only 2,048 (32.1%) of all admitted stroke patients underwent an MRI examination within 1 week of the onset of stroke. A further 1,339 (62.0%) were excluded because they (1) refused to participate or were lost to follow-up; (2) had a history of psychiatric or neurologic disorders; (3) had severe comorbid medical conditions, such as multi-functional organs failure, cancer or severe systemic infection; (4) had severe aphasia, auditory or visual impairments; (5) deceased before the 15-month assessment; (6) had a recurrent stroke during the 15-month follow-up; and (7) were not of Chinese ethnicity.

The study protocol was approved by the Joint Chinese University of Hong Kong New Territories East Cluster Clinical Research Ethics Committee. All participants gave written informed consent.

### Demographic and Clinical Data

Within 1 week of the index admission (T0), demographic (age, sex, and year of education) and clinical data on vascular risk factors (smoking, history of hypertension, diabetes mellitus, hyperlipidaemia, stroke, atrial fibrillation, and ischemic heart disease) were collected. Baseline stroke severity was assessed with the National Institutes of Health Stroke Scale (NIHSS) (Brott et al., 1989) by a nurse from the Stroke Unit. Physical and cognitive functions and level of social support were assessed by a research assistant with the Barthel Index (BI) (Barthel, 1965), the Mini-Mental State Examination (MMSE) (Chiu et al., 1994), and the Lubben Social Network Scale (LSNS) (Lubben, 1988), respectively at T1, T2, and T3.

### Study Outcomes

The primary study outcome was PDS at either T1, T2, or T3. All patients were screened for PDS with the 15-item Geriatric Depression Scale (GDS) by a psychiatrist (W-KT). Clinically significant PDS suspect for an ongoing depressive illness was defined by a score 7 or above on the GDS (Tang et al., 2004a). The 15-item GDS has good sensitivity (89%) and specificity (73%) in screening for PDS in a Hong Kong stroke population (Tang et al., 2004a).

### Neuroimaging

All patients were scanned on a 1.5-T MRI system (Sonata; Siemens Medical Systems Inc., Germany) with diffusion weighted, gradient echo, T1- and T2-weighted and fluid attenuated inversion recovery sequences. The MRI parameters were described in detail elsewhere (Liang et al., 2017). A qualified neurologist (YL) blind to all demographic and clinical information reviewed the MRI data on SVD markers, while another qualified neurologist (Y-KC) conducted the measurement on infarcts. The numbers and volumes of acute and old infarcts were recorded. The details of the scoring method on ‘SVD burden’ were described earlier (Liang et al., 2017). Briefly, based on a validated scale (Staals et al., 2014), the presence of either lacune, cerebral microbleeds, extensive WMH, or moderate to severe enlarged perivascular spaces was awarded one point. Summing the above four subscores produced a total SVD score ranging from 0 to 4. The inter-rater reliability of the MRI characteristics was determined by comparing 30 randomly selected MRI scans rated separately by YL and Y-KC. The inter-rater kappa coefficient values were 0.76 for lacune; 0.63 for periventricular WMH, 0.62 for deep WMH; 0.78 for cerebral microbleeds, 0.80 for enlarged perivascular spaces; and 0.66 for both acute and old infarcts.

### Statistical Analysis

SPSS Statistics, Version 20.0 software (IBM) was used for the statistical analysis. Data are presented as mean ± standard deviation (SD), median (interquartile range, IQR), or proportions, as appropriate. The normality of the data was tested with the Shapiro–Wilk test. To ascertain the associations between SVD burden and PDS during the 15-month follow-up, generalized estimating equation (GEE) models were constructed by including patients attending both the first (T1) and the last (T3) follow-ups (*n* = 563). The summed and individual SVD scores were entered the multivariate GEE models, adjusting for demographic, clinical and imaging characteristics. The level of significance was set at 0.05 (two-tailed).

## Results

### Characteristics of the Study Sample

A total of 563 patients were enrolled at both T1 and T3; 146 (20.5%) patients defaulted at follow-up. **Table 1** lists the characteristics of the study sample and the patients who defaulted. The stroke severity of the study sample was lower than that of patients excluded from the study [NIHSS scores of 3 (1–5) vs. 4 (1–11); *p* < 0.001; data not shown].

**Table 1:** Comparison of demographic, clinical and MRI characteristics between the study sample and the patients withdrawn from the study.

	Included	Withdrawn
	(*n* = 563)	(*n* = 146)
**Demographics**		
Age, years, mean ±*SD*	67.0 ± 10.2	67.1 ± 11.4
Female sex, *n* (%)	230 (40.9)	52 (35.6)
Education, years, mean ±*SD*	6.7 ± 4.2	6.7 ± 5.1
**Vascular risk factors, *n* (%)**		
Current or previous smoker	214 (38.0)	63 (42.9)
Hypertension	364 (64.7)	98 (67.6)
Hyperlipidemia	240 (42.6)	52 (35.9)
Diabetes mellitus	159 (28.2)	44 (30.3)
Previous stroke	56 (9.9)	19 (13.1)
Ischemic heart disease	26 (4.6)	8 (5.5)
Atrial fibrillation	24 (4.3)	16 (11.0)
**Stroke characteristics**		
NIHSS on admission, median (IQR)	3 (1–5)	3 (1–5)
Volume of acute infarcts, ml, mean ± SD	3.2 ± 9.6	3.5 ± 9.1
Number of acute infarcts, mean ±*SD*	1.3 ± 2.2	1.1 ± 1.1
Old infarcts, *n* (%)	114 (20.2)	58 (39.7)
**Location of acute infarcts, *n* (%)**		
Cortical	96 (17.1)	31 (21.2)
Subcortical white matter	173 (30.7)	36 (24.7)
Deep	127 (22.6)	31 (21.2)
Infratentorial	80 (14.2)	30 (20.5)
**SVD individual markers**		
Lacune, *n* (%)	113 (20.1)	28 (19.2)
Cerebral microbleeds, *n* (%)	76 (13.5)	29 (19.9)
Perivascular WMH, median (IQR)	1 (1–2)	1 (1–2)
Deep WMH, median (IQR)	1 (1–2)	1 (1–2)
Enlarged perivascular spaces, median (IQR)	1 (1–2)	1 (0–2)
**SVD burden**		
SVD score, median (IQR)	1 (0–2)	1 (0–2)
SVD score = 0, *n* (%)	248 (44.0)	57 (39.0)
SVD score = 1, *n* (%)	134 (23.8)	30 (20.5)
SVD score = 2, *n* (%)	103 (18.3)	36 (24.7)
SVD score = 3, *n* (%)	54 (9.6)	15 (10.3)
SVD score = 4, *n* (%)	24 (4.3)	8 (5.5)
**Clinical assessments**		
Social support: LSNS, mean ±*SD*	28.3 ± 8.4	28.5 ± 8.7
**PDS, *n* (%)**		
T1	103 (18.3)	20 (13.7)
T2	53 (11.6)	–
T3	69 (12.3)	–
**Severity of PDS: GDS, median (IQR)**		
T1	2 (1–5)	2 (1–4)
T2	2 (1–4)	–
T3	2 (1–4)	–
**Functional independence: BI, mean ± *SD***		
T1	19.1 ± 2.1	19.1 ± 1.9
T2	19.0 ± 2.3	–
T3	19.1 ± 2.1	–
**Cognitive function: MMSE, mean ± *SD***		
T1	26.9 ± 3.1	26.5 ± 3.4
T2	27.1 ± 3.2	–
T3	26.7 ± 3.7	–

*BI, Barthel Index; GDS, Geriatric Depression Scale; IQR, interquartile range; LSNS, Lubben Social Network Scale; MMSE, Mini–Mental State Examination; MRI, Magnetic Resonance Imaging; NIHSS, National Institutes of Health Stroke Scale; PDS, poststroke depressive symptoms; SD, standard deviation; SVD, small vessel disease; WMH, white matter hyperintensities*.

The demographic, clinical and imaging characteristics are shown in **Table 1**. The study sample had a mean age of 67.0 ± 10.2 years; 40.9% were female. The stroke severity was mainly mild to moderate with a median NIHSS score of 3 (IQR, 1–5); 44.0% of the sample had no marked SVD (SVD score = 0) while the median SVD score was 1 (IQR, 0–2). Levels of social support (LSNS: 28.3 ± 8.4), functional independence (BI: 19.1 ± 2.1) and cognitive functions (MMSE: 26.9 ± 3.1) were relatively high. The level of PDS was low with a median GDS score of 2 across the three assessments. The frequency of clinically meaningful PDS at T1, T2, and T3 was 18.3% (103), 11.6% (53), and 12.4% (69), respectively.

### Association Between SVD and PDS

The results of the multivariate GEE model are shown in **Table 2**. The combined SVD score (OR: 1.30, 95% CI: 1.07–1.58; *p* = 0.010) was a significant predictor of PDS over the 15-month follow-up. None of the four individual SVD markers (lacune: *p* = 0.720; cerebral microbleeds: *p* = 0.066; WMH: *p* = 0.430; enlarged perivascular spaces: *p* = 0.369) was significantly associated with PDS in the multivariate GEE models (data not shown).

**Table 2:** Multivariate GEE model on the association between PDS and baseline characteristics.

Significant predictors^a^	OR (95%CI)	*p*
**Time points of assessments**		
T1	Reference	
T2	0.60 (0.42–0.85)	0.004
T3	0.61 (0.45–0.82)	0.001
**Sex**		
Male	Reference	
Female	1.80 (1.02–3.17)	0.042
**Smoking**		
Non-smoker	Reference	
Current or previous smoker	1.75 (1.06–2.90)	0.029
Social support (LSNS)	0.96 (0.93–0.98)	<0.001
**SVD burden (SVD score)**	**1.30 (1.07–1.58)**	**0.010**
Number of acute infarcts	1.11 (1.02–1.20)	0.014
Functional independence (BI)	0.87 (0.81–0.92)	<0.001

*BI, Barthel Index; CI, confidence interval; GEE, generalized estimating equation; LSNS, Lubben Social Network Scale; OR, odds ratio; PDS, poststroke depressive symptoms; SVD, small vessel disease*.

^a^*All the predictors listed here were adjusted for assessment time points, age, sex, social support, vascular risk factors, stroke severity, location, number and volume of acute infarcts, old infarcts, SVD burden, cognitive function, and functional independence*.

### Other Predictors of PDS

Other significant predictors of PDS were female sex (OR: 1.80, 95% CI: 1.02–3.17, *p* = 0.042); smoking (OR: 1.75, 95% CI: 1.06–2.90, *p* = 0.029); number of acute infarcts (OR:1.11, 95% CI: 1.02–1.20, *p* = 0.014); time of assessment at T2 (OR: 0.60, 95% CI: 0.42–0.85, *p* = 0.004) and T3 (OR: 0.61, 95% CI: 0.45–0.82, *p* = 0.001) compared with T1; higher level of social support (OR: 0.96, 95% CI: 0.93–0.98, *p* < 0.001); and functional independence (OR: 0.87, 95% CI: 0.81–0.92, *p* < 0.001) (**Table 2**).

## Discussion

The main finding of this study is that SVD burden is associated with clinically meaningful PDS over a 15-month follow-up period in patients with mild to moderate acute ischemic stroke. To the best of our knowledge, this was the first longitudinal study that examined the relationship between SVD burden and PDS after stroke.

It is of note that the SVD burden rather than any single SVD marker was associated with PDS. Higher SVD burden is associated with risk of depression 3 months after lacunar stroke (Zhang X. et al., 2017). The current study extended this finding by conducting a 15-month follow-up to ascertain the long-term impact of the baseline SVD burden on PDS. All subtypes of ischemic stroke were included because lacunar strokes only account for 25% of ischemic strokes (Kolominsky-Rabas et al., 2001). Total SVD burden has been suggested to be a better predictor of stroke outcomes, including mortality (Song et al., 2017), disability (Arba et al., 2017a), stroke recurrence (Lau et al., 2017), and poor quality of life (Liang et al., 2017), than a single SVD marker. The findings of this study lend further support to the hypothesis that chronic vascular burden in the brain is a critical neuroanatomical factor in depression in older adults (Santos et al., 2009b).

The underlying mechanisms linking SVD burden to poststroke PDS are poorly understood. Functional disability and cognitive impairment may be mediating factors as they confer the highest risk for PSD (Towfighi et al., 2017). SVD lesions reduce the brain’s ‘reserve’ against vascular insults and its plasticity (Mok et al., 2017). Stroke patients with more severe SVD are prone to have larger infarct volume (Helenius et al., 2016), impaired reperfusion (Arba et al., 2017b), and recurrent stroke (Lau et al., 2017). Consequently, disability and cognitive decline were more likely to occur among stroke survivors with severe SVD burden (Huijts et al., 2013; Arba et al., 2017a). However, after adjustment for acute infarcts, stroke severity, functional independence, and cognition in this study, SVD burden remained a significant predictor of PDS. This suggests that SVD burden might contribute to the development of PDS via other paths. From a network viewpoint, the integrity of both the structural and functional global network is the key prerequisite for recovery from stroke (Lim and Kang, 2015) and emotional modulation (Gong and He, 2015). SVD leads to the disruption of white matter integrity of frontal-subcortical circuit which is thought to be critical in the pathogenesis of depression (van Uden et al., 2015). Reduced global structural network efficiency arising from SVD has recently been shown to be associated with depression (Xie et al., 2017).

The pathomechanism of PSD is not yet clarified. In addition to the ‘vascular depression hypothesis’ as mentioned above, evidence is emerging to support the notion that inflammation could also play a role in the development of PSD (Popa-Wagner et al., 2014; Becker, 2016). Higher level of inflammatory markers was recently found to be predictor of PSD (Cheng et al., 2017) and anti-inflammatory treatment has shown promising results in reducing risk of PSD (Wium-Andersen et al., 2017). Furthermore, SVD burden was found to be associated with systemic lupus erythematosus, a systemic inflammatory disease (Wiseman et al., 2016). Enlarged perivascular spaces, one of the SVD markers, was proposed as an MRI marker of neuroinflammatory activity and was associated with lesions in multiple sclerosis (Wuerfel et al., 2008). The above findings suggest that SVD burden could be an epiphenomenon of the accumulating inflammatory processes.

Medical comorbidities are the ‘silent contributors’ to stroke outcomes (Sandu et al., 2015). Intracranial atherosclerosis and diabetes were recently reported to be risk factors of early-onset and late-onset PSD, respectively (Chen et al., 2016; Zhang Y. et al., 2017); smoking, as also demonstrated in this study, was associated with PDS. It is suggested that these comorbidities join SVD to promote a chronic proinflammatory state and fuel acute neuroinflammation in the event of acute stroke (Di Napoli et al., 2012; Sandu et al., 2015). The aggravated neuroinflammation, in turn, causes the downstream neurodegeneration, neuronal apoptosis, and impaired neuroplasticity (Leonard and Maes, 2012), disrupt the depression-related neurocircuits (Loubinoux et al., 2012), and eventually result in depressive symptoms or other unfavorable outcomes (Di Napoli et al., 2012). Taken together, PSD or PDS could be viewed as a consequence of a complex interplay between stroke, pre-stroke comorbidities, and neuroinflammation (Sandu et al., 2015).

Other predictors of PDS in the present study were female sex, lower levels of functional independence and social support, smoking, the time of the assessment, and the number of acute infarcts. Female sex, lower level of functional independence and social support have been consistently reported as risk factors of PSD (Towfighi et al., 2017). Smoking also increases the risk of PSD in minor stroke (Shi et al., 2015).

In this study, the risk of PDS was decreasing during the 15-month follow-up. A one-year follow-up study reported that the prevalence of depression decreased from 18.8% at 1 month to 14.6% (Bour et al., 2010). A similar decline in PSD from 6.7% at 3 months to 5.1% at 1 year has also been reported (Kulkantrakorn and Jirapramukpitak, 2007). The number of acute infarcts predicted PDS in the present study, which is consistent with the finding that more severe lacunar infarcts are associated with PSD (Santos et al., 2009a; Wu et al., 2014).

The major strengths of this study are its prospective design, large sample size and its relatively long 15-month follow-up after the index stroke. The first major limitation is that this study mainly included mild to moderate stroke patients. This selection bias reduces the generalizability of the results, including the relationship of SVD burden and PDS in acute ischemic stroke. Second, the lack of comprehensive clinical assessment of depression limits the clinical implications of the study, although PDS also have detrimental effects on stroke outcomes (Towfighi et al., 2017). Nevertheless, the GDS is a reliable tool to detect PDS in this stroke population and has been shown to be useful for screening PDS in Hong Kong elderly stroke patients with good sensitivity and specificity (Tang et al., 2004a,b). A systemic review has also confirmed that the GDS could detect milder PDS in the elderly (Burton and Tyson, 2015). The third limitation is the lack of etiological differentiation of ischemic stroke due to the limited clinical information. Hence, the study could not clarify the differential impact of SVD burden on PDS with respect to the subtypes of acute ischemic stroke. Fourth, about a quarter of eligible patients were lost to follow-up. To offset this limitation, GEE analysis was used to handle missing data. Fifth, this was an observational study without comprehensive investigation of the inflammatory markers or comorbidities. Moreover, we are aware that ideally the assessment of imaging data should be conducted by two raters despite of moderate to good interrater agreements, however, this study failed to provide confirming assessments by a second rater due to the limited manpower. Finally, the 15-month follow-up could not draw conclusions about the association between SVD burden and longer-term clinical course of PDS.

## Conclusion

SVD burden was associated with clinically significant PDS over a 15-month follow-up in patients with mild to moderate acute ischemic stroke. The findings lend further support to the hypothesis of vascular depression suggesting that accumulating microvascular lesions are critical for the development of depressive symptoms. Measurement of SVD burden might facilitate the early identification of patients at risk of PDS after mild to moderate acute ischemic stroke. Multimodal imaging studies from a network perspective would be useful to clarify the underlying mechanisms linking SVD to PDS. Investigations of the impact of SVD burden on the effectiveness of antidepressant treatment in PDS are also warranted.

## Authors Contributions

W-KT and YL made substantial contributions to the conception and design of the study. YL, Y-KC, VC-TM, D-FW, and WC-WC made substantial contributions to the acquisition of data. YL and Y-KC made substantial contributions to the analysis of data. VM, D-FW, WC-WC, GSU, H-JK, and W-KT contributed to the interpretations of data. YL drafted the first version of the manuscript. All the authors revised the draft for intellectual content, gave their final approval of the final version for publication, and agreed to be accountable for all aspects of the work in ensuring that questions related to the accuracy or integrity of any part of the study are appropriately investigated and resolved.

## Conflict of Interest Statement

The authors declare that the research was conducted in the absence of any commercial or financial relationships that could be construed as a potential conflict of interest.

## Abbreviations

BI: Barthel Index
CI: confidence interval
GDS: Geriatric Depression Scale
GEE: generalized estimating equation
IQR: interquartile ranges
LSNS: Lubben Social Network Scale
MMSE: Mini–Mental State Examination
MRI: Magnetic Resonance Imaging
NIHSS: National Institutes of Health Stroke Scale
OR: odds ratio
PDS: poststroke depressive symptoms
PSD: poststroke depression
SD: standard deviations
SVD: small vessel disease
WMH: white matter hyperintensities.
